# Identification of Time-Series Pattern Marker in Its Application to Mortality Analysis of Pneumonia Patients in Intensive Care Unit

**DOI:** 10.3390/jpm14080812

**Published:** 2024-07-31

**Authors:** Suhyeon Lee, Suhyun Kim, Gayoun Koh, Hongryul Ahn

**Affiliations:** 1 Division of Data Science, The University of Suwon, Hwaseong-si 16419, Republic of Korea; happy_shsh@suwon.ac.kr (S.L.); myksh0903@suwon.ac.kr (S.K.); gayoun0@suwon.ac.kr (G.K.); 2 DS&ML Center, The University of Suwon, Hwaseong-si 16419, Republic of Korea

**Keywords:** time-series pattern marker, electronic health record, mortality analysis, machine learning, deep learning, pneumonia

## Abstract

Electronic Health Records (EHRs) are a significant source of big data used to track health variables over time. The analysis of EHR data can uncover medical markers or risk factors, aiding in the diagnosis and monitoring of diseases. We introduce a novel method for identifying markers with various temporal trend patterns, including monotonic and fluctuating trends, using machine learning models such as Long Short-Term Memory (LSTM). By applying our method to pneumonia patients in the intensive care unit using the MIMIC-III dataset, we identified markers exhibiting both monotonic and fluctuating trends. Specifically, monotonic markers such as red cell distribution width, urea nitrogen, creatinine, calcium, morphine sulfate, bicarbonate, sodium, troponin T, albumin, and prothrombin time were more frequently observed in the mortality group compared to the recovery group throughout the 10-day period before discharge. Conversely, fluctuating trend markers such as dextrose in sterile water, polystyrene sulfonate, free calcium, and glucose were more frequently observed in the mortality group as the discharge date approached. Our study presents a method for detecting time-series pattern markers in EHR data that respond differently according to disease progression. These markers can contribute to monitoring disease progression and enable stage-specific treatment, thereby advancing precision medicine.

## 1. Introduction

Electronic health records (EHRs) are a type of big data in the healthcare and medical field, storing patients’ medical and health information [1]. As a result of decades-long efforts to build EHR datasets, substantial large-scale EHR datasets have been released, such as the Medical Information Mart for Intensive Care II (MIMIC-II) [2] and MIMIC-III in 2015 [3]. Since then, researchers have conducted various data-driven studies on topics such as patient survival prediction, disease diagnosis, disease prognosis, multimodal data integration, and generative language model application using these EHR datasets [4,5,6,7,8].

EHRs are a crucial source for precision or personalized medicine. The U.S. National Human Genome Research Institute defines precision medicine (generally considered analogous to personalized medicine or individualized medicine) as “an innovative approach that uses information about an individual’s genomic, environmental, and lifestyle information to guide decisions related to their medical management” [9]. A prime example of personalized medicine is targeted cancer therapy. This therapy divides cancer patients into subgroups based on genetic mutations and applies targeted treatments selectively, tailored to the mutation type of each subgroup [10]. The factors used for distinguishing these subgroups are known as markers, and various molecular markers have been developed for each type of cancer, including colorectal cancer [11], breast cancer [12], and lung cancer [13]. The personalized medicine approach is increasingly feasible in broader clinical settings by combining phenotypic data from EHRs with genomic data, enabling selective treatments for distinct patient groups [14].

This paper explores the research topic of developing methods to detect medical markers from EHR big data. Here, `markers’ refer to indicators closely associated with a target disease or condition (e.g., mortality from a specific disease, adverse drug reaction), used to classify patients according to the disease/condition’s diagnosis or prognosis, and they are also known as risk factors [15,16,17,18]. These EHR markers can be utilized for personalized medicine by identifying phenotypic cohorts based on the EHR markers and strategically applying specialized treatments to these specific groups [14]. For example, the Simplified Acute Physiology Score II (SAPS II) combines physiological and demographic variables, derived from EHR data, to determine mortality risk, aiding in personalized care in intensive care unit (ICU) [19].

Recently, with the emergence of EHR big data, research that excavates markers through data analysis and algorithms has been progressing [15,16,17,18]. Methods of detecting markers from EHR data typically involve dividing the data into two groups (e.g., patients vs. healthy individuals or non-survivors vs. survivors) and identifying variables that exhibit differences between these two groups. Statistical models or machine learning models are commonly used in this process of finding variables [20]. The Cox and logistic regression models are representative statistical models used for marker detection. For instance, Wang et al. [21] conducted a Cox regression analysis on elderly patients with heart failure to explore the relationship between the systemic inflammation response index (SIRI) and the mortality rate and ICU admission days in heart failure, proposing SIRI as a marker to predict all-cause mortality in this population. Similarly, Zhao et al. [22] performed multivariate logistic regression analysis on sepsis patients, identifying the following as independent prognostic factors for septicemia: age, blood urea nitrogen (BUN), hemoglobin, platelet count, partial plasma thromboplastin time, international normalized ratio, white blood cell count, minimum potassium, renal function impairment, hepatic function impairment, cardiovascular function impairment, and respiratory function impairment. Among these, they suggested that platelet count functions as a marker for sepsis based on a Kaplan-Meier analysis.

Additionally, various machine learning methods are frequently employed in marker detection. For instance, Alsinglawi et al. [23] conducted a Shapley additive explanation (SHAP) analysis based on a random forest model to derive important features related to the hospitalization period of lung cancer patients, such as temperature, emergency admissions, glucose, and respiratory rate. Hong et al. [24], on the other hand, employed gradient boosting machine learning and step-wise feature selection techniques to identify 11 significant variables for predicting survival in pediatric critical care.While they initially identified 397 variables, they demonstrated that using these selected 11 variables for prediction did not significantly reduce predictive accuracy compared to using all 397 variables.

Existing studies employing methods like Cox, logistic regression, SHAP in random forests, and gradient boosting have successfully identified significant markers from EHR data. However, these methods have a critical limitation: they do not account for the dynamic nature of disease progression, which is a prominent feature in EHR data. In ICU settings, the variability and rapidity of disease progression are more pronounced, with patients’ health potentially deteriorating quickly over a short period. In such scenarios, where diseases evolve dynamically, markers are activated differently at various times, depending on the mechanisms with which they are associated. Some markers become noticeable in the early stages of disease development, offering vital opportunities for timely intervention. In contrast, others may only become apparent during advanced stages of deterioration, indicating varying disease pathways and levels of severity.

In this study, we propose a method for detecting markers considering temporal characteristics of EHR big data. The aim of our method is to identify markers from EHR big data that respond at different times during the progression of a disease, such as those reacting early or later. To achieve the goal, we categorized the temporal patterns into two distinct types of responses: monotonic and fluctuating trend patterns. Monotonic trend pattern markers demonstrate consistent responses throughout the entire course of the disease, from its early to late stages. In contrast, fluctuating trend pattern markers show minimal or weak responses in the initial stages of the disease but become significantly responsive in its later stages. Our method, applied on MIMIC-III EHR data for pneumonia patients, successfully identified specific markers corresponding to these patterns: monotonic pattern markers such as red cell distribution width (RDW), BUN, and creatinine as shown in Figure 4A and fluctuating pattern markers such as dextrose in sterile water (DSW), sodium polystyrene sulfonate, free calcium, and glucose as illustrated in Figure 5A,C. If validated through further empirical research, these findings could significantly contribute to categorizing pneumonia subtypes based on the disease stage, thereby enhancing the precision of personalized medicine for critically ill patients.

The paper is structured as follows. In Section 2, we explore related works on variable selection methods in machine learning. Section 3 explains the data and processing. Our proposed method for detecting time-series markers in EHR data is detailed in Section 4. In Section 5, we present the experiments and results, applying the proposed method to the MIMIC-III pneumonia patient mortality dataset to identify time-series pattern markers. Finally, Section 7 discusses the medical relevance of the identified markers to pneumonia and the technical characteristics of our approach.

## 2. Background

This section summarizes the background methodologies used in the proposed approach: the time-series algorithms and the variable importance methods for exploring medical markers.

### 2.1. Time-Series Deep Neural Network Algorithms

Deep neural network algorithms are a type of machine learning algorithm known for their ability to vary the model structure, resulting in the development of various neural network architectures for different types of data [25]. Several deep neural network models have been developed for modeling time-series data, including recurrent neural network (RNN), long short-term memory (LSTM), gated recurrent unit (GRU), and transformer models. RNN [26] summarizes past information in a neural network to influence present predictions for sequential data. However, RNNs struggle to handle long sequences due to the long-term dependency problem. LSTM [26] overcomes this problem of RNNs by utilizing input, output, and forget gates, maintaining long-term memory through a cell state structure. GRU [27] is an evolution of LSTM that simplifies the cell state and output gate, combining them into update and reset gates for a more streamlined model. Additionally, the transformer model [28] differs from RNN, LSTM, and GRU by processing sequential data non-sequentially, using a self-attention mechanism to learn context by considering the relationships between elements within the input sequence. These time-series artificial neural network models have exhibited excellent performance in various prediction problems within recent EHR big data, such as early septic shock diagnosis prediction [29], patient subtyping [30], survival prediction [31], and readmission prediction [32].

### 2.2. Explainable AI and Variable Importance

Explainable artificial intelligence (XAI) is a field of artificial intelligence(AI) research that aims to explain the decision-making process of machine learning in a way that people can understand [33]. XAI emerged to address the limitation of black-box AI algorithms, which fail to clearly present the rationale behind the predictions made by AI systems [34]. Through various approaches such as visualization, knowledge extraction, influence methods, and example-based explanation, XAI attempts to explain the reasoning behind predictions [35]. As a category of XAI, variable importance (or feature importance (FI)) is a method that quantifies the importance of variables in a model’s predictions [36]. By indicating the importance scores of variables predicted by machine learning models, variable importance indirectly explains the model’s prediction process. In EHR data, variable importance can be utilized as a marker detection method by presenting the importance of each medical variable. The following subsections introduce representative techniques for variable importance, such as FI, permutation importance (PI), and SHAP.

### 2.3. FI of Tree Ensemble

Tree ensembles, such as random forests [37] and gradient boosting [38], are a popular class of machine learning algorithms that train multiple decision trees and make predictions by voting from the decision trees. One important aspect of tree ensembles is their ability to produce FI, the relevance scores of each feature to a given prediction task. Gini importance [37] is a representative way to compute the FI in a tree ensemble and it consists of three steps.

First, it focuses on a decision tree *t* in the tree ensemble and computes the node importance for each node in the decision tree. Let *n* be one of the nodes in the decision tree where the node divides the samples with the proportion of $p_{c}$ for *c* class. Then, the Gini impurity of the node *n* is as follows:

(1) $$G\left(n\right)=1-\sum_{c\in all\;classes}p_{c}^{2}$$

Continuously, let the node *n* be split into left/right child nodes $n_{L}$ and $n_{R}$, and $w_{n}$ be the weighted number of samples reaching node *n*. Then, the node importance of *n* is as follows:

(2) $$I\left(n\right)=w_{n}G\left(n\right)-w_{n_{L}}G\left(n_{L}\right)-w_{n_{R}}G\left(n_{R}\right)$$

Second, it calculates the importance of each feature in the decision tree. The $F_{i}$ of *i* is as follows:

(3) $$F_{i}^{\left(0\right)}=\frac{\sum_{n\in nodes\;split\;by\;feature\;i}I\left(n\right)}{\sum_{k\in all\;nodes}I\left(k\right)}$$

Then, the $F_{i}$ is normalized across the features in the decision tree *t*:

(4) $$F_{i}^{\left(t\right)}=\frac{F_{i}^{\left(0\right)}}{\sum_{j\in all\;features}F_{j}^{\left(0\right)}}$$

Lastly, it produces the final $F_{i}$ as the mean of $F_{i}$ across the decision trees in the ensemble, where *T* is the total number of trees:

(5) $$F_{i}=\frac{\sum_{t\in all\;trees}F_{i}^{\left(t\right)}}{T}$$

### 2.4. PI

PI [37] is a technique used in machine learning to determine the importance of features or variables in a given model. It is a model-agnostic method, meaning it can be applied to any machine learning algorithm regardless of its underlying structure or complexity.

The basic idea behind PI is to shuffle or permute the values of a particular feature in a dataset and observe the effect on the model’s performance. The feature is then ranked based on the reduction in performance caused by the shuffling. Features that significantly reduce performance when shuffled are considered more important, while those that have little effect on performance are considered less important.

Let *X* be a dataset, *Y* be a target vector, *f* be a trained machine learning model, s(Y,f(X)) be a performance score (e.g., accuracy or *f*1 score) of the target vector *Y* and a prediction vector f(X), and $X_{j}$ be a dataset by permuting feature *j* in the data. Then, the PI of a feature *j* is as follows:

(6) $$P_{j}=s\left(Y,f\left(X\right)\right)-s\left(Y,f\left(X_{j}\right)\right)$$

### 2.5. SHAP

SHAP [39] is a method for interpreting machine learning models that helps to explain how individual features contribute to model predictions. SHAP is based on the concept of Shapley values, which were originally developed in the context of cooperative game theory to allocate the value or profit generated by a group of players to individual players.

In the machine learning context, Shapley values represent the contribution of each feature to a given prediction. The SHAP approach calculates the difference between the predicted value for a given instance with all its features and the predicted value when a specific feature is removed or set to a reference value. This calculation is repeated for all possible combinations of features, and the average of all these differences is calculated. This average is the Shapley value for that feature, which represents that feature’s contribution to the prediction.

Let *F* be the set of all features, F/i be the set of all features except a feature *i*, *S* be a subset of F/i, $X_{S\cup \{i\}}$ be the dataset including the features S∪{i}, $f_{X_{S\cup \{i\}}}$ be a trained machine learning model using the dataset $X_{S\cup \{i\}}$, and $f_{X_{S\cup \{i\}}}\left(X_{S\cup \{i\}}\right)$ be the prediction by the model $f_{X_{S\cup \{i\}}}$ for the dataset $X_{S\cup \{i\}}$. Then, the Shapley value of feature *i* is as follows:

(7) $$\begin{array}{c} \phi_{i}=\sum_{S\subset F/i}K_{S}\left[f_{X_{S\cup \{i\}}}\left(X_{S\cup \{i\}}\right)-f_{X_{S}}\left(X_{S}\right)\right],\\ \mathrm{where}\;K_{S}=\frac{|S|!(|F|-|S|-1)!}{|F|!} \end{array}$$

## 3. Data and Processing

This section introduces the MIMIC-III dataset used in the study and explains how we processed it to construct a machine learning dataset for identifying time-series pattern markers in pneumonia patients.

### 3.1. MIMIC-III Dataset

The MIMIC-III dataset [3] is a collection of time-series EHRs tracking medical events such as diagnoses, tests, and prescriptions for 46,520 patients who stayed in the ICU at Beth Israel Deaconess Medical Center from 2001 to 2012. Individuals who have completed training provided by PhysioNet and obtained permission are allowed partial access to the MIMIC-III dataset. The dataset comprises 26 separate CSV files structured into tables, categorized based on information about patients, admissions, diagnoses, tests, prescriptions, and so on. It has contributed significantly to advancements in medical AI research, including studies related to patient recovery, disease prediction, and length of hospital stay [40,41,42].

### 3.2. Machine Learning Dataset Processing

We structured a time-series machine learning dataset to predict the mortality of pneumonia patients by processing the 26 CSV files from the MIMIC-III dataset. The machine learning dataset is divided into explanatory variable dataset *X* and predictive variable dataset *Y*.

Below, we explain the process of generating the machine learning dataset involving three steps: pneumonia patient selection, explanatory variable dataset generation, and predictive variable dataset creation.

Pneumonia patient selection. We identified patients diagnosed with pneumonia-related diseases using International Classification of Diseases, Ninth Revision (ICD-9) codes, specifically 486 (Pneumonia, organism unspecified), 5070 (Pneumonitis due to inhalation of food or vomitus), and 48,241 (Methicillin-susceptible pneumonia due to Staphylococcus aureus), from the PATIENTS file, resulting in a total of 7727 pneumonia cases.

Explanatory variable dataset generation. The explanatory variable dataset *X* is a binary 3D tensor representing the values of *F* medical variables for *N* pneumonia patients across *T* dates, denoted as $X=\left\{x_{i,t,f}\right\}\in {\left\{1,0\right\}}^{N\times T\times F}$. Here, *N* represents the number of patients, *T* denotes the number of time points, and *F* indicates the number of medical event variables. *X* is generated through the following process.

- We select a single patient among the 7727 pneumonia patients, referred to as the *i*th patient.
- We investigate the death or discharge date of the *i*th patient from the ADMISSIONS file and set that date as the reference date (D0) for that patient. We then extract medical event data for 10 days before the reference date (i.e., ($D_{-10}∼D_{-1}$ where, *T* = 10) from the LABEVENTS, PROCEDURE EVENTS, and PRESCRIPTIONS files. LABEVENTS refers to results of experimental test such as blood tests, PROCEDURE EVENTS denotes data related to the patient’s procedures, and PRESCRIPTIONS represents the patient’s prescribed medications.
- We perform binary encoding based on the type of medical event. For variables in the LABEVENTS file, if the result of any lab test (e.g., blood glucose test) is outside the normal range, the value is binary encoded as 1; otherwise, it is encoded as 0. For variables in the PROCEDURE EVENTS and PRESCRIPTIONS files, if any procedure or prescription is ongoing that day, the value is binary encoded as 1; otherwise, it is encoded as 0.
- We repeat this process for all pneumonia patients and exclude medical events that never have a value of 1, reducing the 4068 types of medical events to 3595 event types.

Through the above process, we constructed a 3D time-series explanatory variable dataset *X* of dimensions ${\left\{1,0\right\}}^{N\times T\times F}$, where *N* = 7727, *T* = 10, *F* = 3595.

Predictive variable dataset creation. The predictive variable dataset *Y* comprises target values representing the death or recovery of pneumonia patients. The target values serve as label information for AI model predictions. For the *i*th patient $P_{i}$, if s/he was discharged due to death on day $D_{0}$, s/he is assigned a value of 1; if s/he recovered and was discharged to a regular ward, s/he is assigned a value of 0, creating the predictive variable dataset $Y_{i}\in \left\{1,0\right\}$ for patient $P_{i}$. Then, extending this to the pneumonia patients *N*, the explanatory variable dataset becomes $Y=\left\{Y_{1},\cdots ,Y_{N}\right\}\in {\left\{1,0\right\}}^{N}$. Thus, we generated the one-dimensional binary vector objective variable dataset *Y* for *N* = 7727 pneumonia patients, with approximately 61% of pneumonia patients in *Y* having died.

**Figure 1:**
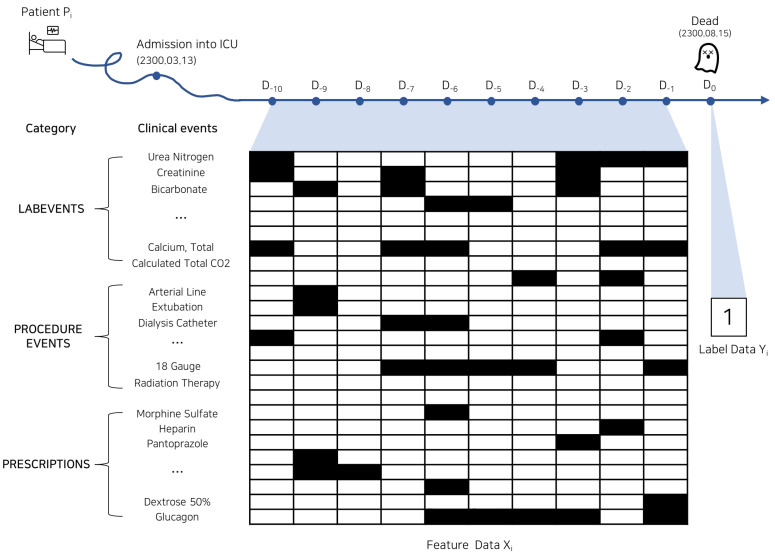
Example of 10-day medical time-series event data for single patient. The horizontal axis represents the reference day ($D_{0}$), which is the date of the patient’s death or discharge, along with the 10 days preceding to the reference day ($D_{-10}∼D_{-1}$). The vertical axis denotes the types of medical events. In the middle of the figure, black and white cells indicate binary values, signifying that a medical event occurred and did not occur, respectively, on that specific date.

Figure 1 visually illustrates an example of time-series data within the machine learning datasets *X* and *Y*, showcasing the time-series data for a pneumonia patient in the 10 days prior to his/her date of death or discharge ($D_{-10}∼D_{-1}$).

## 4. Methods

**Figure 2:**
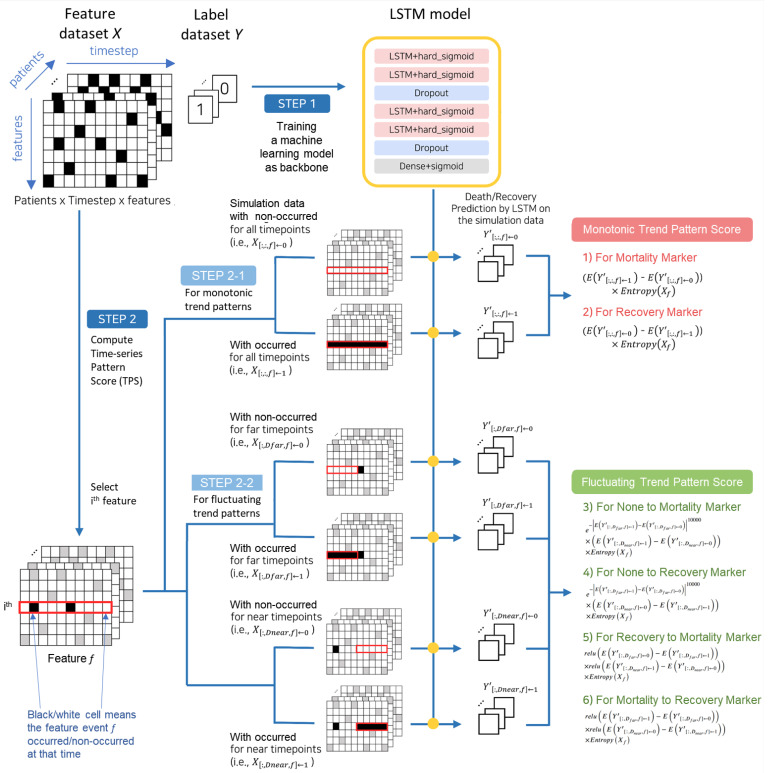
Procedure of suggested method of detecting time-series pattern marker in EHR data. Firstly, the proposed method takes an EHR machine learning dataset and a time-series trend pattern as input. It then calculates and produces TPS TPS(f) for each medical event variable $f\in F_{1},\cdots ,F_{F}$. It then completes the following two steps: (1) training a time-series machine learning model and (2) computing pattern scores through data simulation.

In this section, we propose a method of detecting time-series pattern markers in EHR data consisting of two steps: (1) training a time-series machine learning model and (2) computing pattern scores through data simulation, illustrated in Figure 2. The proposed method takes an EHR machine learning dataset and a time-series trend pattern (e.g., a pattern in which there is a consistently higher occurrence rate in the mortality group compared to the recovery group over time) as input. Then, it computes and outputs time-series pattern scores (TPSs) TPS(f) for each of the medical event variables $f\in F_{1},\cdots ,F_{F}$. The pattern score TPS(f) measures how closely the values of the variable *f* in the data align with the input time-series trend pattern. In this paper, we propose six types of input trend patterns, which are described in detail in STEPS 2-1 and 2-2. Among the six trend patterns, two are monotonic trend patterns, showing consistently higher occurrence rates in one group over the other group. The other four are fluctuating trend patterns, including one in which there is no past difference but then higher occurrence rates emerge in one group and one in which one group has higher past occurrence rates, but then occurrence rates increase in the other group over time.

### 4.1. Step 1: Training Time-Series Machine Learning Model

In the first step, we train a time-series machine learning model to predict patient mortality. This trained model is used as the backbone model in the subsequent time-series pattern detection step. Our time-series pattern detection method is a model-agnostic method. In other words, it is not limited to a specific model as a backbone model; rather, it can use various types of time-series neural network models such as RNN, LSTM, and GRU, as backbone models. After comparing the mortality prediction accuracy of several machine learning models in the MIMIC-III EHR dataset, we selected the LSTM model as the backbone model due to its superior accuracy (see the Section 5). The top part of Figure 2 illustrates the LSTM model architecture used in our pattern marker detection method. We employed binary cross-entropy as the loss function and the Adam optimizer. Additionally, early stopping was applied to prevent overfitting, setting the maximum number of training epochs to 300.

### 4.2. Step 2-1: Calculating Monotonic Trend Pattern Scores through Data Simulation

In this step, the pattern scores for monotonic trend patterns are computed, as depicted in the bottom part of Figure 2. Monotonic trend patterns refer to those occurring consistently at a high frequency within a single group over time. For instance, in an EHR dataset involving pneumonia patients who either died or recovered, a monotonic trend pattern for mortality indicates a consistent occurrence at a high frequency within the mortality group, while a monotonic trend pattern for recovery indicates a consistent occurrence at a high frequency within the recovery group.

The monotonic trend pattern score TPS(f) of a medical event $f\in \left\{F_{1},\cdots ,F_{F}\right\}$ is calculated as follows.

First, by transforming the data values of the medical event *f* from the original explanatory variable data *X*, we obtain two types of simulated data: occurrence simulation data $X_{[:,:,f]\leftarrow 1}$ and non-occurrence simulation data $X_{[:,:,f]\leftarrow 0}$.

- $X_{[:,:,f]\leftarrow 1}$, the occurrence simulation data, represents the values of variable *f* changed to “occurred” values (1) for all patients (:) at all time points (:) in the original data *X*.
- $X_{[:,:,f]\leftarrow 0}$, the non-occurrence simulation data, represents the values of variable *f* changed to “not occurred” values (0) for all patients (:) at all time points (:) in the original data *X*.

After generating the occurrence and non-occurrence simulation data, we predict the class label for these datasets using the backbone model *M* trained on the original data in STEP 1. This yields the predicted simulation values ${Y^{\prime }}_{f\leftarrow 1}=M\left(X_{f\leftarrow 1}\right)$ and ${Y^{\prime }}_{f\leftarrow 0}=M\left(X_{f\leftarrow 0}\right)$. Ultimately, the monotonic pattern score TPS(f) for medical event *f* regarding mortality is computed by taking the product of the difference between the averages of ${Y^{\prime }}_{f\leftarrow 1}=M\left(X_{f\leftarrow 1}\right)$ and ${Y^{\prime }}_{f\leftarrow 0}=M\left(X_{f\leftarrow 0}\right)$, and the entropy of $X_{f}$.

(8) $$TPS\left(f\right)=\left(E\left({Y^{\prime }}_{f\to 1}\right)-E\left({Y^{\prime }}_{f\to 0}\right)\right)\times Entropy\left(X_{f}\right)$$

The defined monotonic pattern marker score holds the following characteristics:

If the medical event *f* is a mortality marker event (i.e., positively correlated with death), the mortality marker score TPS(f) will have a large positive value. For events positively correlated with death, the average trend of patient mortality, $E({Y^{\prime }}_{f\to 1})$, increases in the predicted results of the occurrence simulation data ${Y^{\prime }}_{f\to 1}$, while the average trend $E({Y^{\prime }}_{f\to 0})$ decreases in the predicted results of the non-occurrence simulation data ${Y^{\prime }}_{f\to 0}$. Consequently, for medical events *f* correlated with mortality markers, $E\left({Y^{\prime }}_{f\to 1}\right)-E\left({Y^{\prime }}_{f\to 0}\right)$ becomes a large positive value.

If the medical event *f* is a recovery marker event (i.e., negatively correlated with death), the mortality marker score TPS(f) will have a small negative value, in contrast to the case of mortality markers.

If the medical event *f* is a commonly occurring event, $Entropy\left(X_{f}\right)$ increases. Therefore, the absolute value of TPS(f) increases (i.e., considering commonly occurring events as important). This is because $Entropy(X_{f})$ in the formula becomes a positive value that increases when the medical event *f* occurs commonly and decreases when it occurs rarely.

Users can assign an appropriate weight to $Entropy(X_{f})$ in the marker score formula to detect either commonly occurring markers or rare markers.

### 4.3. Step 2-2: Calculating Fluctuating Trend Pattern Scores through Data Simulation

In this step, pattern scores for trend-changing patterns over time are computed. Certainly, while there may exist complex trend-changing patterns with various variations, complicated trends might have limited practical applicability in the field. Therefore, this paper presents four medically meaningful types of trend-changing patterns: (1) none to mortality, (2) none to recovery, (3) recovery to mortality, and (4) mortality to recovery. By dividing the entire period of 10 days ($D_{-10},\cdots ,D_{-1}$) into two subperiods—$D_{far}=D_{-10},\cdots ,D_{-6}$ as the distant past and $D_{near}=D_{-5},\cdots ,D_{-1}$ as the recent past, the pattern marker score for the four types of trend patterns can be represented by the following equation.

(1) None to mortality pattern. A pattern in which there is no difference in the distant past, but the occurrence rate increases in the mortality group in the recent past.

(9) $$\begin{array}{cc} PS(f) & ={e^{-\left|E\left({Y^{\prime }}_{[:,D_{far},f]\leftarrow 1}\right)-E\left({Y^{\prime }}_{[:,D_{far},f]\leftarrow 0}\right)\right|}}^{10,000}\\ \; & \times \left(E\left({Y^{\prime }}_{[:,D_{near},f]\leftarrow 1}\right)-E\left({Y^{\prime }}_{[:,D_{near},f]\leftarrow 0}\right)\right)\\ \; & \times \;Entropy\left(X_{f}\right) \end{array}$$

(2) None to recovery pattern. A pattern in which there is no difference in the distant past but the occurrence rate increases in the recovery group in the recent past.

(10) $$\begin{array}{cc} PS(f) & ={e^{-\left|E\left({Y^{\prime }}_{[:,D_{far},f]\leftarrow 1}\right)-E\left({Y^{\prime }}_{[:,D_{far},f]\leftarrow 0}\right)\right|}}^{10,000}\\ \; & \times \left(E\left({Y^{\prime }}_{[:,D_{near},f]\leftarrow 0}\right)-E\left({Y^{\prime }}_{[:,D_{near},f]\leftarrow 1}\right)\right)\\ \; & \times \;Entropy\left(X_{f}\right) \end{array}$$

(3) Recovery to mortality pattern. A pattern in which the occurrence rate is higher in the recovery group in the distant past but increases in the mortality group in the recent past.

(11) $$\begin{array}{cc} PS(f) & =relu\left(E\left({Y^{\prime }}_{[:,D_{far},f]\leftarrow 0}\right)-E\left({Y^{\prime }}_{[:,D_{far},f]\leftarrow 1}\right)\right)\\ \; & \times \;relu\left(E\left({Y^{\prime }}_{[:,D_{near},f]\leftarrow 1}\right)-E\left({Y^{\prime }}_{[:,D_{near},f]\leftarrow 0}\right)\right)\\ \; & \times \;Entropy\left(X_{f}\right) \end{array}$$

(4) Mortality to recovery pattern. A pattern in which the occurrence rate is higher in the mortality group in the distant past but increases in the recovery group in the recent past.

(12) $$\begin{array}{cc} PS(f) & =relu\left(E\left({Y^{\prime }}_{[:,D_{far},f]\leftarrow 1}\right)-E\left({Y^{\prime }}_{[:,D_{far},f]\leftarrow 0}\right)\right)\\ \; & \times \;relu\left(E\left({Y^{\prime }}_{[:,D_{near},f]\leftarrow 0}\right)-E\left({Y^{\prime }}_{[:,D_{near},f]\leftarrow 1}\right)\right)\\ \; & \times \;Entropy\left(X_{f}\right) \end{array}$$

## 5. Experiments and Results

### 5.1. Comparison of Mortality Prediction Accuracy among Machine Learning Models

**Figure 3:**
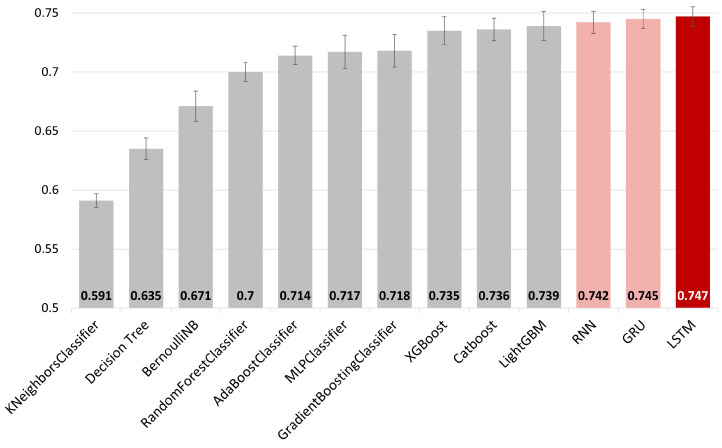
AUROC accuracies for predicting mortality in pneumonia patients. The horizontal axis represents machine learning models categorized by color: non-time-series model (gray), time-series model (light red), and LSTM (dark red). The vertical axis represents AUROC.

We compared the accuracy of non-time-series machine learning models (KNN, decision tree, Bernoulli NB, random forest, Adaboost, MLP, gradient boosting, XGBoost, CatBoost, LightGBM) and deep learning-based time-series models (LSTM, RNN, GRU) in predicting the mortality of pneumonia patients in the MIMIC-III EHR dataset. For the latter, we used the time-series data *X* as described in the Data and Processing section for training and prediction. For the former, a non-time-series dataset was constructed for training and prediction by performing a union operation along the time axis on the time-series data *X* to eliminate the temporal dimension, resulting in the following non-time-series dataset $X^{\prime }=\mathrm{UNION}\left(X,\mathrm{axis}\;=\;t\right)\in {1,0}^{N\times F}$. To generalize the comparison results of the models, we performed 10-fold cross-validation with an 8:2 training-to-test data ratio, deriving the average area under the receiver operating characteristic (AUROC) as the final accuracy metric. The comparison results indicated that time-series models exhibited higher accuracy than non-time-series models, with LSTM demonstrating the highest accuracy among the former (Figure 3).

### 5.2. Monotonic Trend Patterns in Mortality and Recovery Markers in Pneumonia Patients

We utilized the LSTM model from the previous section’s results as the backbone model to apply our marker detection method, resulting in the calculation of TPS ($TPS_{F_{1},\cdots ,F_{3595}}$) for a total of 3595 medical event variables concerning mortality pneumonia patients and recovery groups among pneumonia patients. From the ordered scores, We selected the top 10 monotonic trend pattern markers for both mortality and recovery. The 10 mortality markers were RDW, urea nitrogen, creatinine, calcium, morphine sulfate, bicarbonate, sodium, troponin T, albumin, and prothrombin time (PT). The 10 recovery markers were heparin, alanine aminotransferase (ALT), pantoprazole, mean corpuscular hemoglobin concentration (MCHC), dextrose 50%, glucagon, potassium chloride, 20-gauge, aspartate aminotransferase (AST), and bisacodyl.

**Figure 4:**
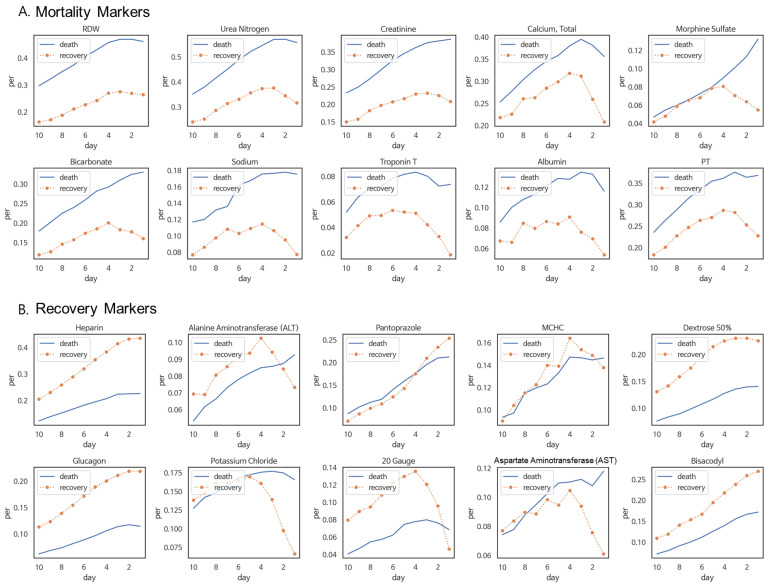
Event occurrence frequency plots for monotonic pattern markers of the mortality and recovery groups among pneumonia patients. (**A**) mortality markers and (**B**) recovery markers, with each marker being selected as the top 10 based on monotonic pattern scores calculated by the proposed time-series marker detection method. The horizontal axis represents the 10 days ($D_{-10}∼D_{-1}$) before the date of death/discharge ($D_{0}$), and the vertical axis indicates the percentage ratio of event occurrence frequency in the mortality group (blue) and recovery group (orange).

Subsequently, we visualized the occurrence rates for the selected mortality and recovery markers (Figure 4). The visualization results showed that for all 10 mortality markers, throughout the time-series period, the frequency of marker occurrences was higher in the mortality group than the recovery group (Figure 4A). Additionally, the recovery markers exhibited a tendency in which, during the time-series period, the frequency of marker occurrences was higher in the recovery group than the mortality group (Figure 4B).

### 5.3. Fluctuating Trend Pattern Markers in Pneumonia Patients

**Figure 5:**
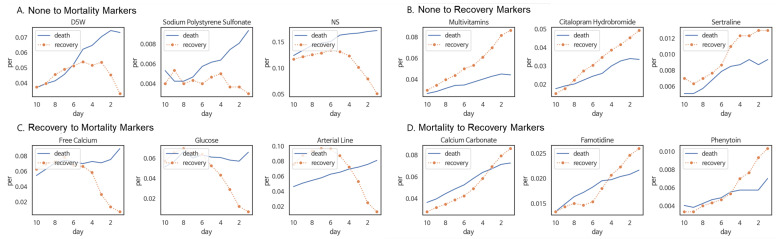
Event occurrence frequency plots for fluctuating pattern markers. (**A**) none to mortality markers (**B**) none to recovery markers (**C**) recovery to mortality markers (**D**) mortality to recovery markers. The horizontal axis represents the 10 days ($D_{-10}∼D_{-1}$) before the date of death/discharge ($D_{0}$), and the vertical axis indicates the percentage ratio of event occurrence frequency in the mortality group (blue) and recovery group (orange).

We applied the proposed method to pneumonia patient data and calculated fluctuating trend pattern marker scores for a total of 3595 medical events. We selected and visualized the top 10 markers for each of the following four specific patterns: one in which there is no difference in the distant past but the occurrence rate increases in the mortality group in the recent past, one in which there is no difference in the distant past, but the occurrence rate increases in the recovery group in the recent past, one in which the occurrence rate is higher in the recovery group in the distant past, but increases in the mortality group in the recent past, one in which the occurrence rate is higher in the mortality group in the distant past but increases in the recovery group in the recent past. Some of the chosen markers exhibited the targeted patterns (Figure 5).

### 5.4. Comparison with Existing Marker Detection Methods

We compared the TPS method with existing machine learning marker detection methods, including the FI, PI, and SHAP methods. Among our time-series pattern detection methods, we included only monotonic trend patterns in the comparison excluding fluctuating trend patterns, as existing variable importance methods do not consider temporal patterns during the marker detection process, making it impossible to compare them with fluctuating trend patterns. Additionally, FI provides positive importance scores without distinguishing between mortality and recovery markers. Therefore, we calculated the importance scores of variables in terms of absolute values, without differentiating between mortality and recovery markers.

Different marker detection methods may yield different results depending on the machine learning model used as the backbone. Therefore, to robustly compare the marker detection methods, we applied multiple machine learning models as backbone models to each marker detection method. For the TPS method, we employed time-series deep learning models (RNN, LSTM, GRU) as backbone models. For variable importance methods, we utilized non-time-series tree ensemble models (decision tree, random forest, Adaboost, gradient boosting, XGBoost, LightGBM, Catboost) as well as time-series deep learning models. However, in the FI method, time-series deep learning models were not utilized as backbone models because FI did not work with them. Additionally, in the PI method, time-series deep learning models were not used as backbone models due to excessively long execution times. As a result, the total number of combinations for the compared variable importance methods and their backbone models was 27, as summarized below.

- TPS with backbone models of RNN, LSTM, GRU
- FI with backbone models of decision tree, random forest, Adaboost, gradient boosting, XGBoost, LightGBM, Catboost
- PI with backbone models of decision tree, random forest, Adaboost, gradient boosting, XGBoost, LightGBM, Catboost
- SHAP with backbone models of decision tree, random forest, Adaboost, gradient boosting, XGBoost, LightGBM, Catboost, RNN, LSTM, GRU

**Figure 6:**
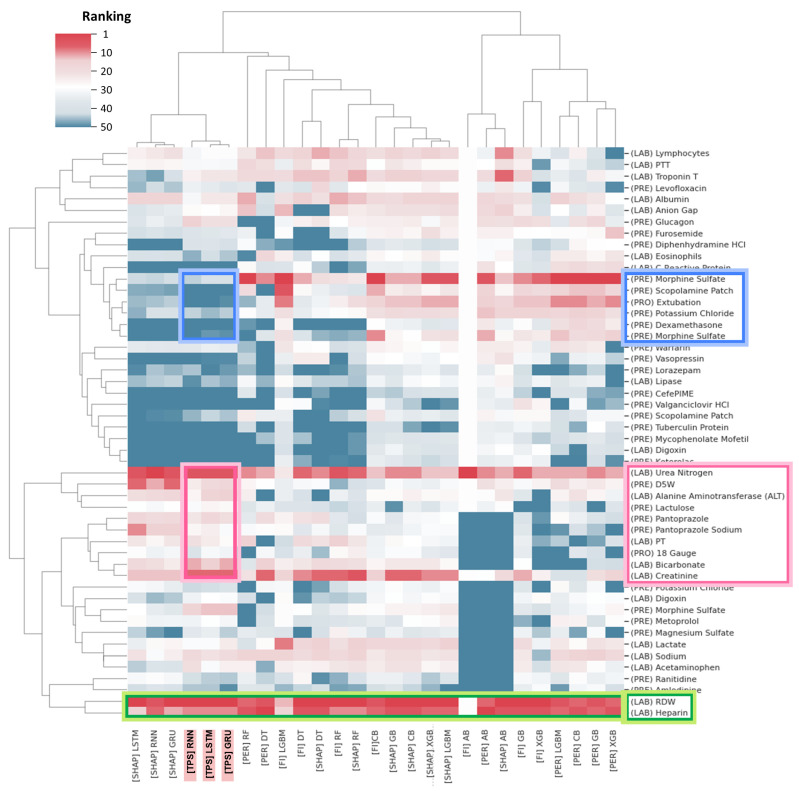
Heatmap visualization of markers identified by various marker detection methods. The horizontal axis represents marker detection methods (TPS, FI, PI) for various machine learning models as backbone models. The vertical axis represents a total of 50 variables, which are the union of the top 10 markers extracted from each marker detection method. The color of the cells indicates the importance ranking of each variable in each method (red for high, blue for low). The variables highlighted by the green box are those commonly ranked among the top variables in both our method and most other machine learning marker detection methods. Those highlighted by the red and blue boxes are distinctively selected as top variables in our method and other machine learning marker detection methods, respectively.

For all 27 results, we extracted the top 10 variables with high absolute importance scores. Afterward, we formed the union variable set using the extracted variables, resulting in a total of 50 variables. For these union variables, we built a heatmap to visualize the importance rankings of each method. In the heatmap, we analyzed variables that were commonly or differentially considered important across our method and existing variable importance methods (Figure 6).

Variables like RDW and heparin were commonly top-ranked across both our method and most other machine learning marker detection methods (highlighted by the green box in Figure 6). However, variables like urea, nitrogen, DSW, ALT, lactulose, pantoprazole, pantoprazole sodium, PT, 18 gauge, bicarbonate, and creatinine were ranked higher in our method (highlighted by the red box in Figure 6), while variables like morphine sulfate, scopolamine patch, extubation, potassium chloride, and dexamethasone were ranked higher in other machine learning marker detection methods (highlighted by the blue box in Figure 6).

## 6. Discussion

### 6.1. Literature Review of Time-Series Pattern Markers of Pneumonia

This section discusses the association between the pattern markers selected through our analysis and pneumonia. The results of the literature review indicate that all 10 selected monotonic mortality trend pattern markers are associated with the occurrence or exacerbation of pneumonia.

- RDW has been identified as a mortality-associated marker in studies involving cardiovascular patients [43] and elderly individuals [44]. Lee et al. [45] investigated the correlation between RDW and mortality in 744 patients with respiratory pneumonia, suggesting it as a prognostic marker for the disease.
- It has been reported that elevated levels of BUN are more frequently found in pneumonia-related deaths [46,47,48]. In a study involving over 1900 pneumonia patients, it was identified as a variable associated with ICU admission and mortality [49].
- Decreased creatinine levels have been reported as a significant factor associated with the occurrence of and mortality from pneumonia in elderly patients undergoing dialysis [50]. A study tracking 121,762 hemodialysis patients for a maximum of 5 years showed that not only serum creatinine reduction but also weight loss, muscle mass reduction, and low weight were associated with higher mortality rates in hemodialysis patients. Furthermore, serum creatinine reduction was reported as a more powerful predictor of mortality than weight loss in hemodialysis patients [51].
- Calcium deficiency was significantly observed in a study comparing 302 pneumonia patients with 300 healthy individuals [52]. Additionally, recent reports on COVID-19 patients indicated a tendency towards low calcium levels, especially in patients with severe illness [53].
- Morphine sulfate, a potent analgesic, is used for pain reduction and relief of respiratory distress symptoms in severely ill patients such as those with advanced cancer [54]. It is also prescribed for severe pneumonia. In a pneumonia case study, it demonstrated a symptom relief effect of 77% for dyspnea in patients with acute exacerbation (AE) of end-stage interstitial pneumonia (IP). [55].
- It has been reported that elevated levels of bicarbonate are significantly associated with the onset and exacerbation of pneumonia. In an experimental comparison of 302 pneumonia patients and 300 healthy individuals, the former group exhibited higher bicarbonate levels (*p* < 0.01) than the latter [52]. Additionally, a study involving 671 ventilator-associated pneumonia (VAP) patients in the ICU found that patients with high levels of serum bicarbonate ions had a higher mortality rate [56].
- A low level of sodium is known to be positively correlated with pneumonia occurrence [57]. Furthermore, several studies have consistently reported a positive relationship between hyponatremia and higher mortality rates in pneumonia [58,59,60,61].
- Troponin T, an indicator that increases when myocardial cells are damaged, is utilized as a marker for predicting myocardial infarction. A correlation between high levels of troponin T and mortality in pneumonia patients has been reported by several studies [17,62,63].
- High levels of albumin have been associated with an increased risk of complications and mortality in pneumonia patients. The research team led by Viasus conducted a study with 3463 community-acquired pneumonia (CAP) patients, revealing an association between albumin levels and the incidence of complications and mortality [64]. Another study led by Lee confirmed the association between a 28-day mortality rate and albumin in patients admitted with high-severity CAP [65].
- The research team led by Tripodi reported that PT, an indicator of blood clotting time, is prolonged in CAP patients [66]. Additionally, an association between prolonged PT and the exacerbation of pneumonia related to COVID-19 has been reported. Wang’s study on 213 confirmed COVID-19 patients indicated prolonged PT in the deceased group [67]. Baranovskii’s research, comparing COVID-19 patients requiring intensive care within 2 weeks of hospitalization with stabilized COVID-19 patients, suggested that PT levels at admission could serve as early prognostic indicators for severe pneumonia [68].

Furthermore, the results of the literature review demonstrated the relationship between the occurrence or exacerbation of pneumonia and the recovery monotonic trend pattern markers or fluctuating trend pattern markers we identified. Among them, those related to blood clotting and blood glucose are discussed below.

- Heparin is a substance with anticoagulant properties commonly prescribed to prevent thrombosis. Heparin prevent blood clotting, leading to delayed PT. However, in severe pneumonia, blood clotting disorders have been observed, and there tends to be an increase in PT [67,68]. Therefore, the use of Heparin, a blood anticoagulant, may need to be avoided in severe pneumonia patients. Further research is needed on blood clotting disorders in severe pneumonia patients and the prescription of heparin.
- Glucagon, a hormone secreted from the pancreas, breaks down glycogen in the liver into glucose, increasing blood sugar levels. Medically, it is used to treat hypoglycemia. A study conducted by Zeng, involving 290 elderly patients with CAP reported that admission blood glucose levels exceeding 11.1 mmol/L were significantly associated with ICU admission and 30-day survival rates [69]. In our results, blood sugar levels were elevated in both deceased and recovering patients, but in recovering patients, blood sugar tended to decrease as the discharge date approached. Consequently, glucagon, which increases blood sugar levels, may be administered more to recovering patients. Further research is needed on the relationship between blood sugar and glucagon in pneumonia patients in ICU.

The time-series pattern markers identified in this study demonstrate associations with both the onset and exacerbation of pneumonia, aligning with findings from previous research. In the following section, we explore the medical implications of these markers and discuss their potential roles in the treatment and management of pneumonia in clinical settings.

### 6.2. Implications about Personalized Medicine

Our findings support the existing literature, underscoring a potential link between the identified time-series pattern markers and pneumonia. This validation enhances the credibility of our approach and lays a groundwork for further investigation into these associations.

Our research unveils new insights into the detailed patterns of pneumonia progression, enriching our understanding of the disease’s dynamics. We have pinpointed two types of markers, each indicative of distinct trends in disease progression over the 10-day period we analyzed:

- Monotonic Trend Markers: These markers exhibit consistent responses throughout the disease course, specifically over the 10-day period from onset to advanced stages, underscoring their potential for early detection of pneumonia.
- Fluctuating Trend Markers: These markers become increasingly significant in the later stages of the 10-day period, potentially indicating a worsening condition.

Utilizing these markers enables clinicians to potentially devise stepwise monitoring and intervention strategies, which are tailored to the progression stage of a patient’s pneumonia. Such an approach could also lead to the development of new indices that enhance existing clinical scores like SAPS II, offering a more comprehensive assessment of a patient’s health status. The proposed scoring system could facilitate the staging of pneumonia into categories of disease severity, guiding the development of customized treatment plans. This method is in line with the principles of personalized medicine, aiming to tailor treatment to the specific progression of an individual’s disease during this critical period.

However, it is crucial to note that these applications are currently theoretical. Implementing these markers in clinical practice for personalized medicine will require extensive empirical research and validation. This should include experimental verification of each marker’s relevance to disease progression, the development of robust scoring systems and classification criteria, and rigorous statistical testing to evaluate the effectiveness of these markers in clinical applications.

### 6.3. Technical Discussion on TPS Method

In this section, we engage in a technical discussion of the data normalization method, the LSTM model, and the proposed TPS approach.

During our data preprocessing stage, we perform the one-hot encoding method to normalize values of various types of variables into 1s and 0s. One-hot encoding offers the following advantages for normalizing and analyzing EHR data.

Firstly, by employing one-hot encoding, we can utilize values labeled by medical experts during the EHR data normalization process. The variables in EHR data are measured from thousands of medical tests, procedures, prescriptions, and diagnoses, exhibiting highly diverse characteristics. Among these, medical test variables generally have numerical values. To accurately normalize them, it is necessary to consider the unique characteristics of each variable, such as the unit of measurement, statistical properties (distribution type, mean, variance), and normal range. Verifying and validating the characteristics of thousands of variables for normalization is time-consuming.

However, in MIMIC-III EHR data, medical experts provide labeled flag values for numerical test variables. This labeling is based on the normal range of information that medical experts already know. If the values fall within the normal range, they are labeled “normal”; otherwise, they are labeled “abnormal”. Therefore, by performing one-hot encoding with “normal” as 1 and “abnormal” as 0 based on the flag values, we can utilize values validated by medical experts during the normalization process. Additionally, since the process of extracting flag values from the MIMIC-III data is computationally straightforward, the normalization process can be automated through programming.

Furthermore, by utilizing one-hot encoding, the values of all variables are transformed into a uniform meaning, enabling the integrated interpretation of variables with different characteristics. In EHR data, there are not only numerical but also categorical variables. Since numerical values and categorical values generally require different approaches in analysis and interpretation, it is challenging to perform integrated analysis in datasets where both types coexist. However, through one-hot encoding, the values of variables can be standardized into 0s and 1s, where 1 represents the occurrence of a medical event variable and 0 denotes its non-occurrence, allowing for a consistent interpretation of the binary meaning of these values. Moreover, we leveraged this binary encoding of values in our data simulation, where changing a value from 0 to 1 indicates the occurrence of the medical variable and changing it from 1 to 0 means non-occurrence. This simulation served as a core principle in our methodology for identifying time-series pattern markers.

On the other hand, one-hot encoding introduces information loss as it simplifies all values into 1s and 0s. Researchers intending to conduct studies and analyses using our TPS method should keep this possibility in mind.

Next, we discuss the role of the LSTM model in our TPS method. The LSTM, a deep neural network specifically optimized for sequential data such as time-series or text, is used as a backbone model in our data simulation process. Its strength lies in its ability to learn temporal contexts, leveraging both recent and more distant past events to inform current decisions. This is accomplished by encapsulating state information up to a specific time point within long-term and short-term memory vectors, and then delivering these vectors to the subsequent prediction stage. Such a distinctive capability allows the LSTM to outperform other machine learning models that are not specialized in time-series data, in terms of prediction accuracy for time-series data [70,71,72,73,74]. Furthermore, In our study on predicting mortality among pneumonia patients, the LSTM demonstrated the highest level of AUROC accuracy, as shown in Figure 3.

In the TPS method, we maintain the internal structure of the LSTM, using it as an ‘AI predictor’ during data simulation. This involves manipulating input data to explore specific temporal patterns. For monotonic patterns, feature values are altered across the entire time range, whereas for fluctuating patterns, modifications are confined to half of the time range. The LSTM simulates the effects of these data alterations on mortality prediction, effectively transmitting past changes to future predictions through its short-term and long-term memory vectors. By measuring the variations in mortality predictions made by the LSTM, we score how changes in predictions align with the intended temporal patterns. The way in which the LSTM transmits manipulated data from earlier to later time points is integral to the TPS data simulations within a time-series context. This explanation offers an intuitive understanding of how LSTM contributes to the TPS method. However, further research is needed to enhance our comprehension of how LSTM models propagate data modifications over time.

Moreover, we discuss the features of the TPS method compared with existing machine learning-based variable importance methods such as FI, PI, and SHAP.

First, our TPS method differs from existing machine learning-based variable importance methods in that it considers trend patterns over time to identify markers. Existing variable importance methods such as FI, PI, and SHAP can only identify markers with high occurrence frequency in specific groups and do not take into account temporal features. In contrast, our TPS method considers the temporal trend changes in the occurrence frequency when identifying markers. As shown in Figure 4 and Figure 5, it can identify markers with trend patterns of consistently high occurrence frequency in one group over time, or markers with changing trends (e.g., being highly frequent in the mortality group in the distant past and, transitioning to a high frequency in the recovery group in the recent past).

The markers identified by the TPS method, considering monotonic trend patterns, exhibited both similarities and differences compared to markers detected by existing machine learning marker detection methods. As seen in Figure 6, RDW and heparin were consistently top-ranking markers in both our TPS and existing machine learning-based methods. Nitrogen, DSW, ALT, lactulose, pantoprazole, pantoprazole sodium, PT, 18-gauge, bicarbonate, and creatinine were markers relatively high-ly ranked only in our method. On the other hand, morphine sulfate, scopolamine patch, extubation, potassium chloride, and dexamethasone were markers high-ly ranked only in existing machine learning marker detection methods. The discovery of common markers between our method and existing methods indicates shared characteristics in marker identification. The distinctive results imply that the methodology developed in this study presents a new type of approach that yields novel results not confined to existing methodologies. Further experimental and statistically rigorous analysis will be necessary to determine the medical significance of the time-series markers we have identified.

### 6.4. Future Works

In this section, we review the limitations of our study and discuss future research directions.

First, our TPS method can be further analyzed using a detailed pneumonia cohort dataset to derive knowledge about segmented pneumonia markers. In our study, we applied the TPS method to identify pneumonia markers showing responses at various times from a cohort dataset of 7751 ICU-admitted pneumonia patients diagnosed with three pneumonia ICD-9 codes (486—Pneumonia, organism unspecified, 5070—Pneumonitis due to inhalation of food or vomitus, 48,241—Methicillin susceptible pneumonia due to Staphylococcus aureus) from the MIMIC-III dataset. The markers identified included those responding in the early stages, such as RDW, BUN, creatinine, calcium, morphine sulfate, bicarbonate, sodium, troponin T, albumin, and PT, and those responding in the later stages, such as DSW, polystyrene sulfonate, free calcium, and glucose. By segmenting the cohort considering different characteristics of pneumonia markers (e.g., regional characteristics, genetic traits, patient numbers, age, types of comorbid diseases) and applying the TPS method, more segmented features of pneumonia markers can be detected. For example, analyzing cohorts of patients with bacterial infectious pneumonia, patients with septic complications, or cohorts of specific age groups can yield specialized and detailed markers related to specific causes of pneumonia.

Second, the generalizability of our TPS method in various conditions can be investigated. The study analyzed 7751 ICU-admitted pneumonia patients with 3595 variables. Future research can explore whether our TPS method works to detect time pattern markers in different cohort characteristics. Characteristics worth exploring include the number of patients, different diseases, regions, demographics, and various types of hospital systems. Among these, the number of patients is directly related to generalizability. In machine learning, the ratio of variables to the amount of data greatly impacts generalizability. Previous research recommends a minimum data-to-variable ratio (n/p) of at least 5 [75]. However, increasing the number of data points (n) in EMR datasets is challenging due to the need for patient disease occurrences. Thus, researching the appropriate n/p ratio and the minimum n at which the TPS method performs adequately would be a practical topic for studying generalizability. Furthermore, the generalizability characteristics of the TPS method can vary depending on the type of disease, region and demographics, and various types of hospital systems. Therefore, applying TPS to cohort datasets with different characteristics and studying the appropriate parameters will provide practical insights into the generalizability of the TPS method.

Third, experimental studies can be conducted to confirm the practical correlation mechanisms of pneumonia time-series markers identified from EMR big data. This study performed a retrospective analysis to find markers showing specific time-series patterns in the collected EMR big data. As future research, a prospective study can confirm the practical disease relevance of retrospectively identified markers. For example, by specifying candidate markers and pneumonia patient cohorts and designing experiments to generate data in a controlled environment, the precise statistical correlation between specific markers and pneumonia can be explored. Furthermore, by designing and conducting biochemical, genetic, and cellular experiments on the role of specific markers in pneumonia progression, the biological mechanisms by which these markers influence pneumonia can be investigated. Conducting statistical and biological experimental validation of these markers can comprehensively confirm their roles, thereby concretely identifying the potential for their use in personalized medicine.

Finally, the technical advancement of our TPS method can be pursued. Specifically, recent transformer models used in time-series models can be leveraged as the backbone of the TPS method. In our task of predicting mortality in ICU pneumonia patients, models considering time structure like RNN, GRU, and LSTM showed better accuracy than machine learning models that did not consider time structure, with LSTM showing the highest accuracy (refer to Figure 3). Other studies have shown that LSTM and GRU models outperform RNNs in sequence data prediction, and as observed in various studies, LSTM and GRU provide improved accuracy for specific problems [76,77,78,79]. Recently, more complex transformer models have been used for time-series data prediction modeling [28,80]. Future research is needed to evaluate the impact on the accuracy and robustness of mortality prediction when using such more complex models. Additionally, proposing modifications or new structures to better adapt time-series models to data simulation is an important methodological research direction for the future.

## 7. Conclusions

In this study, we introduced a method for identifying time-series pattern markers in EHR data. Our approach takes EHR data and a specified time-series trend pattern as input, producing a score that indicates how closely each variable in the data aligns with the given time-series pattern. By applying our method to MIMIC-III data from pneumonia patients in the ICU, we successfully identified time-series pattern markers in both deceased and surviving patient groups. Visualization of the frequency of these identified time-series pattern markers confirmed their alignment with the query time-series trend patterns. Furthermore, the existing literature provided evidence supporting the association of these markers with the occurrence and exacerbation of pneumonia. We anticipate that our method will contribute to the healthcare field by facilitating the exploration of medical markers based on time-series patterns.

## Data Availability

The study uses the MIMIC-III clinical dataset, which is available at https://physionet.org/, accessed on 25 January 2023. The source code for data processing, modeling, and analysis is available at https://github.com/limeorange/MIMIC_Research, accessed on 25 January 2023.

## Abbreviations

The following abbreviations are used in this manuscript:

AE	acute exacerbation
AI	artificial intelligence
ALT	alanine aminotransferase
AST	aspartate aminotransferase
AUROC	Area Under the Receiver Operating Characteristics
BUN	Blood Urea Nitrogen
CAP	community-acquired pneumonia
EHR	Electronic Health Record
FI	Feature Importance
GRU	Gated Recurrent Unit
ICD-9	International Classification of Diseases, Ninth Revision
ICU	Intensive care unit
IP	interstitial pneumonia
LSTM	Long Short-Term Memory
MCHC	mean corpuscular hemoglobin concentration
MIMIC-III	Medical Information Mart for Intensive Care III
PI	Permutation Importance
PT	prothrombin time
RDW	red cell distribution width
RNN	Recurrent Neural Network
SAPS II	Simplified Acute Physiology Score II
SHAP	Shapley Additive Explanation
SIRI	systemic inflammation response index
TPS	Time-Series Pattern Score
VAP	ventilator-associated pneumonia
XAI	eXplainable Artificial Intelligence
